# Melon diversity on the Silk Road by molecular phylogenetic analysis in Kazakhstan melons

**DOI:** 10.1270/jsbbs.22030

**Published:** 2023-04-25

**Authors:** Katsunori Tanaka, Mitsuhiro Sugiyama, Gentaro Shigita, Ryoma Murakami, Thanh-Thuy Duong, Yasheng Aierken, Anna M Artemyeva, Zharas Mamypbelov, Ryuji Ishikawa, Hidetaka Nishida, Kenji Kato

**Affiliations:** 1 Faculty of Agriculture and Life Science, Hirosaki University, 3 Bunkyo, Hirosaki, Aomori 036-8561, Japan; 2 Institute of Vegetable and Floriculture Science, National Agriculture and Food Research Organization (NARO), 360 Kusawa, Ano, Tsu, Mie 514-2392, Japan; 3 Graduate School of Environmental and Life Science, Okayama University, 1-1-1 Tsushima Naka, Kita-ku, Okayama, Okayama 700-8530, Japan; 4 Faculty of Agronomy, University of Agriculture and Forestry, Hue University, 102 Phung Hung Street, Hue City, Vietnam; 5 Center for Hami Melon, Xinjiang Academy of Agricultural Sciences, Urumqi 830091, China; 6 All-Russian Institute of Plant Genetic Resources on the name of N.I.Vavilov (VIR), 42-44 Bolshaya Morskaya Street, Saint Petersburg 190000, Russian Federation; 7 Kazakhstan Research Institute of Potato and Vegetable Growing LLC, 1 Nauryz Street, Karasay, Almaty 040917, Kazakhstan

**Keywords:** Central Asia, *Cucumis melo*, diversity, genetic resources, on-farm conservation

## Abstract

To uncover population structure, phylogenetic relationship, and diversity in melons along the famous Silk Road, a seed size measurement and a phylogenetic analysis using five chloroplast genome markers, 17 RAPD markers and 11 SSR markers were conducted for 87 Kazakh melon accessions with reference accessions. Kazakh melon accessions had large seed with exception of two accessions of weedy melon, Group Agrestis, and consisted of three cytoplasm types, of which Ib-1/-2 and Ib-3 were dominant in Kazakhstan and nearby areas such as northwestern China, Central Asia and Russia. Molecular phylogeny showed that two unique genetic groups, ^ST^Ia-2 with Ib-1/-2 cytoplasm and ^ST^Ia-1 with Ib-3 cytoplasm, and one admixed group, ^ST^I_AD_ combined with ^ST^Ia and ^ST^Ib, were prevalent across all Kazakh melon groups. ^ST^I_AD_ melons that phylogenetically overlapped with ^ST^Ia-1 and ^ST^Ia-2 melons were frequent in the eastern Silk Road region, including Kazakhstan. Evidently, a small population contributed to melon development and variation in the eastern Silk Road. Conscious preservation of fruit traits specific to Kazakh melon groups is thought to play a role in the conservation of Kazakh melon genetic variation during melon production, where hybrid progenies were generated through open pollination.

## Introduction

Melon (*Cucumis melo* L.) is a widely consumed crop from the *Cucumis* genus, similar to cucumber (*Cucumis sativus* L.), West Indian gherkin (*Cucumis anguria* L.) and horned melon (*Cucumis metuliferus* E. Mey. Ex Naudin 1859), and has various uses as a fruit or vegetable and in medicine, and aroma therapy (Pitrat 2016). Melon originated in Africa and parts of India and has been distributed to wide geographical areas across a long history of melon utilization of more than four thousand years (Sebastian *et al.* 2010, Walters 1989, Zohary and Hopf 2000). During long history of utilization, melon fruit traits, such as fruit size, fruit sutures, epicarp color, pulp color, pulp bitterness, sourness, and sweetness, have been improved to suit human needs (Liu *et al.* 2020, Wang *et al.* 2021, Zhao *et al.* 2019). Based on phenotypic traits including fruit traits, at least 19 horticultural groups have been proposed: Agrestis, Kachri, Chito, Tibish, Acidulus, Momordica, Conomon, Makuwa, Chinensis, Flexuosus, Chate, Dudaim, Chandalak, Indicus, Ameri, Cassaba, Ibericus, Inodorus, and Cantalupensis (Pitrat 2016). Numerous local varieties, market classes and melons with traits that do not fit to known horticultural groups exist worldwide (Stepansky *et al.* 1999) because of the ability to generate hybrid progenies via crossing. Classification of Central Asian melons has been conducted with morphological and physiological traits (Filov 1960, Pangalo 1950). However, analyses of phylogenetic relationships and population structure was rare and limited to Iranian melons and northwestern Chinese melons, even though Central Asian melons are important genetic resources considering their long history along the famous Silk Road (Aierken *et al.* 2011, Raghami *et al.* 2014, Zhang *et al.* 2017).

Central Asia is thought to be one of the centers of the crop (Vavilov 1992), and is an important point where the trade of goods, flow of people and introduction of cultures has occurred. During the long history of Central Asia, animals and plants such as horses, apples, and apricots have undergone domestication and improvement via genomic changes (Duan *et al.* 2017, Librado *et al.* 2017, Liu *et al.* 2019). Central Asian melons were historically appreciated in Iran, India and China, and their cultivation developed along melon trades (Mavlyanova *et al.* 2005). This region has a desert climate with little rain, low humidity, high day and low night air temperatures, and strong and persistent sunshine, of and is considered to be suitable for sweet melon cultivation (Aierken *et al.* 2011, Mavlyanova *et al.* 2005, Zhang *et al.* 2017).

Kazakh melons are cultivated mainly in southern area under irrigation from rivers, such as the Syr Darya, and sold on the road side and in commercial markets together with melons imported from Uzbekistan and Kyrgyzstan. Melons are eaten raw, and sweetness is generally preferred in Kazakhstan. Fruit appearance, such as fruit size, fruit shape, fruit surface morphology and color, and flesh traits are recognized by consumers and are useful for Kazakh melon classification. Kazakh melons are comprised of include various groups, including known horticultural groups Agrestis, Ameri, Cantalupensis, Cassaba, Chandalak, and Inodorus and local melon groups Zard and Zurbek. Some melon groups are also found in Uzbekistan and northwestern China (Aierken *et al.* 2011, Mavlyanova *et al.* 2005), indicating relationships between Kazakh melons and those of nearby areas. By assessing phenotypic and molecular data, we can provide an overview of their phylogenetic relationships and population structure.

Random amplified polymorphic DNA (RAPD) markers can be used to evaluate plant materials with simple experimental procedures and instruments. The low reproducibility of RAPD markers is often caused by reagent, experimental instrument, and experimenter. Nevertheless, molecular evaluation was successful for melons from wide geographical ranges based on two series of RAPD markers: one from Staub *et al.* (2000) and another from Tanaka *et al.* (2007). These two series of RAPD markers can be used for the high-confidence molecular evaluation of melons (Aierken *et al.* 2011, Dhillon *et al.* 2007, Duong *et al.* 2021, Luan *et al.* 2008). Simple sequence repeat (SSR) markers are more polymorphic compared with RAPD markers and have higher reproducibility (Aierken *et al.* 2011, Duong *et al.* 2021, Staub *et al.* 2000). Useful SSR markers have been constructed for genotyping melons from a wide geographical area (Fernandez-Silva *et al.* 2008, Fukino *et al.* 2007, Kong *et al.* 2007). These SSR markers have been used to classify the diversity within melons (Chikh-Rouhou *et al.* 2021, Raghami *et al.* 2014, Tzitzikas *et al.* 2009, Zhang *et al.* 2017). Two standard SSR marker sets have also been constructed and applied to the classification of melons from a wide geographical area (Duong *et al.* 2021, Escribano *et al.* 2012, López-Sesé *et al.* 2003, Nhi *et al.* 2010). Another useful marker for melon classification is developed in the chloroplast genome, which is transmitted maternally (Havey *et al.* 1998). Sequence polymorphisms in chloroplast genome are useful to uncover the origin and phylogenetic relationships of melons (Endl *et al.* 2018, Sebastian *et al.* 2010). For large-scale screening, PCR-based markers such as cleaved amplified polymorphic sequence (CAPS) markers and derived cleaved amplified polymorphic sequence (dCAPS) markers were developed by Nhi *et al.* (2010) and Shigita *et al.* (2023). These markers were used to classify melons into three cytoplasm types, Ia, Ib-1/-2 and Ib-3, which were designated based on the analysis of melons from a wide geographical area (Tanaka *et al.* 2013). Here, molecular markers were used to reveal the phylogenetic relationships and population structure of Kazakh melons.

In this study, we measured seed size and genotyped chloroplast DNA, SSR, and RAPD marker loci across 87 melon accessions from southern Kazakhstan. We analyzed their phylogenetic relationship and population structure, and compared with 115 reference accessions from Aierken *et al.* (2011) to evaluate development and diversity in Kazakh melon.

## Materials and Methods

### Plant materials and DNA extraction

A total of 87 melon accessions from Kazakhstan (*Cucumis melo* L.) were used in this study (Supplemental Table 1). These accessions were collected in four provinces, Almaty, Zhambyl, South Kazakhstan, and Kyzylorda, in southern Kazakhstan in 2011. Each accession was originated from a single fruit which had typical fruit appearance and fruit size within melon fruits found at the collected site, such as market and farmland. Based on fruit characteristics, as shown in Supplemental Table 2, and communication with Mr. Mamypbelov Z. about Kazakh melon classification, 63 of 87 melon accessions were classified into six known horticultural groups proposed by Pitrat (2016), including Agrestis, Ameri, Cantalupensis, Cassaba, Chandalak, and Inodorus and two local variety groups, Zard and Zurbek (Supplemental Fig. 1). Group Zard was classified into six subgroups: Basvaldy, Guliabi, Kalaysan, Kara Guliabi, Sali Guliabi, and Zard. The remaining 24 accessions had admixed fruit phenotypes of known horticultural groups and were classified as the unknown melon group. The seeds from a single fruit collected are maintained as genetic resources at Okayama University and were used for seed size measurement and DNA genotyping in this study.

A total of 115 melon accessions, including two Kazakh melon accessions, were used as reference accessions for an analysis of seed size data and molecular data from Aierken *et al.* (2011) (Supplemental Table 1). Thus, a total of 202 melon accessions were included in the data analysis.

### Seed size measurement

The length and width of 10 randomly selected seeds per accession were measured by using a hand-held CD-AX caliper (Mitutoyo, Japan); these seeds were harvested from a single mature fruit. Based on the average three-seed length, each accession was classified as a large-seed melon (≥9.00 mm) or a small-seed melon (<9.00 mm) after Akashi *et al.* (2002).

### Cytoplasm type by chloroplast genome marker

For one plant from each accession, genomic DNA was extracted from a single ten-day-old seedling after Murray and Thompson (1980) with minor modifications. Chloroplast genome type, assigned as the cytoplasm type, was determined based on an insertion or deletion in the region of *psbC* to *trnS* (InDel1 assigned by Tanaka *et al.* 2013) and four single nucleotide polymorphisms in the regions of *trnK* to *matK* (SNP2), *rpl16* to *rpl14* (SNP18), *ndhF* to *rpl32* (SNP19), and *ndhA* intron 1 (SNP30). InDel1 marker, ccSSR-7, was developed by Chung and Staub (2003). SNP30 was detected by cleaved amplified polymorphic markers (CAPS), and the remaining three SNPs by derived cleaved amplified polymorphic sequence markers (dCAPS), as described in Aierken *et al.* (2011) and Shigita *et al.* (2023) (Supplemental Table 3). The genotyping was performed according to Aierken *et al.* (2011) for InDel1 by ccSSR-7 marker and SNP18 by dCAPS marker and to Shigita *et al.* (2023) for the remaining three markers. The cytoplasm type of each melon accession was determined based on the genotypes of five chloroplast sequence polymorphisms, according to Tanaka *et al.* (2013).

### Genotyping by RAPD and SSR markers and their statistical analysis

Sixteen RAPD and 11 SSR markers were selected for their reproducibility and ability to detect polymorphisms in Kazakh melon accessions (Aierken *et al.* 2011, Nhi *et al.* 2010, Tanaka *et al.* 2007). RAPD marker bands were scored as 1 for a positive band and 0 for a null band. For SSRs, marker fragments were scored based on their size from smallest (1) to largest (2–7, depending on the marker). From these data, calculations for the number of effective alleles (Ne), observed heterozygosity (Ho) and expected heterozygosity (He) and AMOVA were performed by using GenALEX v6.503 (Peakall and Smouse 2012). The gene diversity and polymorphic information content (PIC) within each group were calculated according to Nei (1973) and Botstein *et al.* (1980), respectively.

### Analysis of phylogenetic relationships among accessions and populations

Phylogenetic analysis was conducted using the data of 202 accessions including Kazakh melon and reference accessions to clarify the genetic relationships in melons between Kazakhstan and neighboring countries. Genetic relationships were also analyzed for 17 melon populations with different horticultural groups or geographic origin as following: seven from Kazakh horticultural melon group Kazakhstan (Ameri, Basvaldy, Cassava, Chandalak, Kara guliabi, Sary guliabi, Zard), two from northwestern China (Ameri, Zard), each one from Iran, Afghanistan, Pakistan, Central Asia and Russia, and Spain, and three from USA (Honeydew, Cassava, Cantalupensis). The genetic distance (GD) among accessions and among populations was calculated as described by Apostol *et al.* (1993) and Nei (1972), respectively. Based on the GD matrix, a dendrogram by using the unweighted pair group method with arithmetic averages (UPGMA) cluster analysis was constructed by using PHYLIP v3.698 program (https://evolution.genetics.washington.edu/phylip.html) and was compared with that constructed by the neighbor-joining (NJ) method. Fixation index (F_ST_) value between melon populations from Kazakhstan and overseas with more than five accessions were calculated by GenALEX v6.503 (Peakall and Smouse 2012).

### Population structure analysis

The model-based software program STRUCTURE v2.3.4 (Pritchard *et al.* 2000) was used to infer population structure for all 202 accessions with a Bayesian approach using the RAPD and SSR marker dataset. The optimal value of K (the number of clusters) was deduced by evaluating K = 1–10 and determined by an admixture model with an allele frequencies correlated model. The length of the burn-in for the Markov chain Monte Carlo (MCMC) iterations was set to 5,000, and data were collected over 5,000 MCMC iterations in each run. Twenty iterations per K were conducted. The optimal value of K was identified using the ad hoc procedure introduced by Pritchard *et al.* (2000) and the method developed by Evanno *et al.* (2005), which were carried out in the online program ‘Structure Harvester’ (Earl and vonHoldt 2012). Data plotting after STRUCTURE simulation was conducted with CLUMPP (Jakobsson and Rosenberg 2007).

## Results

### Seed size measurement

Kazakh melons had a seed length greater than 9.76 mm, classifying them as large-seeded melons, with the exception of two small-seeded melon accessions from Group Agrestis with a seed length of 5.76 mm (Supplemental Fig. 2).

### Cytoplasm type by chloroplast genome marker

The DNA fingerprints of the CAPS and dCAPS markers and insertion or deletion markers corresponded with the respective nucleotide sequences in five regions of the chloroplast genome (Supplemental Table 4). Eighty-seven Kazakh melon accessions examined were classified into three cytoplasm types: 2 accessions into Ia, 52 into Ib-1/-2, and 33 into Ib-3. The Ib-1/-2 and Ib-3 cytoplasm types were dominant in Kazakhstan.

### Genotyping by RAPD and SSR markers and their statistical value

Sixteen RAPD markers and 11 SSR markers generated a total of 92 alleles in the 202 melon accessions examined, of which 70 alleles were detected in 87 Kazakh melon accessions (Table 1). The number of alleles per SSR locus ranged from two to four in the Kazakh melon accessions, for which no unique alleles were obtained. The expected heterozygosity (He) ranged from 0 to 0.763, corresponding to the polymorphic information content results (r = 0.758). The mean He was higher in SSR markers than in RAPD markers in the Kazakh melon accessions (0.390 and 0.133, respectively). Since those of reference accessions were 0.580 and 0.383, respectively, the mean He was lower in Kazakh melon. Heterozygosity within SSR loci was observed in the Kazakh melon accessions, although the Ho values ranged from 0 to 0.213 (Mean: 0.055) and were lower than the He values (Range: 0.118 to 0.634, Mean: 0.390).

### Phylogenetic relationships among accessions

**Fig. 1.**
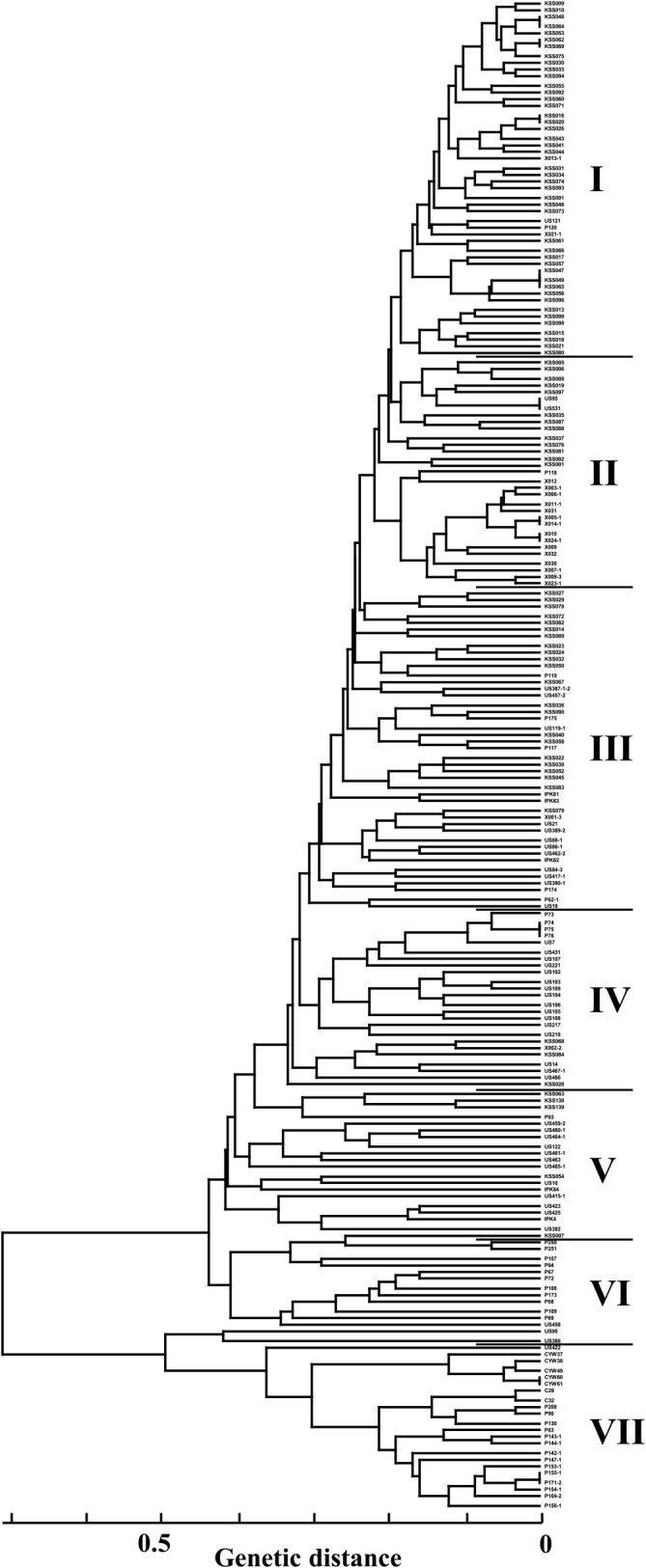
Genetic groups inferred by the distance-based method using UPGMA analysis.

The pairwise genetic distances between 202 melon accessions were calculated from the RAPD and SSR data and ranged from 0 to 0.936, with an average of 0.393 (data not shown). The GDs calculated by combining the RAPD and SSR data was also related to the GDs calculated by RAPD data (r = 0.966, P < 0.01) and SSR data (r = 0.874, P < 0.01) alone. The GDs calculated from RAPD data alone and from SSR data alone significantly correlated (r = 0.718, P < 0.01). The genetic relationships between the 202 melon accessions were visualized by UPGMA cluster analysis based on the genetic distance calculated from frequencies of both RAPD marker and SSR alleles (Fig. 1). The 202 melon accessions were grouped into seven groups, which were assigned as Cluster I to Cluster VII.

### Population structure

**Fig. 2.**
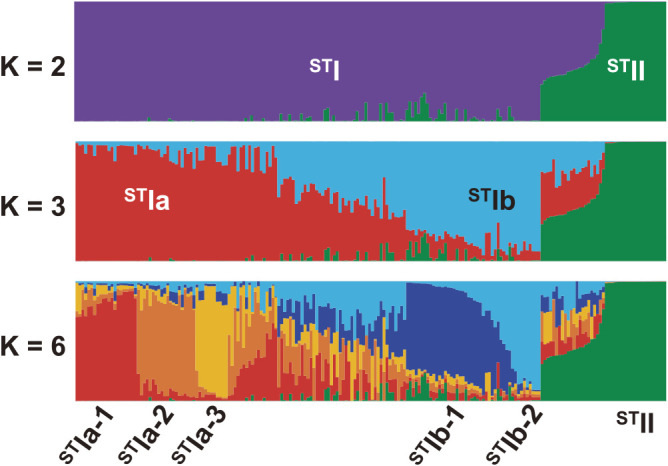
Genetic groups inferred by model-based clustering method. For each accession, group is determined by the membership probability (Q > 0.70) and is shown on the bottom side as “^ST^Ia-1”, “^ST^Ia-2”, and “^ST^Ia-3”, etc.

The LnP (D) value and Delta K value suggested the presence of two groups in the 202 accessions; 181 accessions were allocated into the two groups designated ^ST^I and ^ST^II, and the remainder in the admixed group ^ST^AD, with assignment probabilities (Q) > 0.70 (Fig. 2). Substructuring under the topmost hierarchy was detected for the accessions in ^ST^I at K = 3 and K = 6. Consequently, using model-based classification, the 202 accessions were divided into ^ST^I (159 accessions), ^ST^II (22) and the admixed group ^ST^AD (21). ^ST^I was separated into five subgroups: ^ST^Ia-1 (21), ^ST^Ia-2 (20), ^ST^Ia-3 (11), ^ST^Ib-1 (26), and ^ST^Ib-2 (10), with three admixed subgroups: ^ST^IAD (44), ^ST^IaAD (17) and ^ST^IbAD (10). This model-based classification was significantly correlated with the distance-based classification by the UPGMA cluster analysis (χ^2^ = 643.83, P < 0.01; Cramer’s V = 0.73, P < 0.01). The combined results of these two classifications provided the following phylogenetic overview: ^ST^Ia located in Clusters I–III showed some divergence from ^ST^Ib which was mainly found in Clusters III–V; ^ST^IAD overlapped with ^ST^Ia and ^ST^Ib; ^ST^II in Cluster VII was grouped alone; and ^ST^AD in Clusters V and VI was an intermediate group between ^ST^I and ^ST^II.

**Fig. 3.**
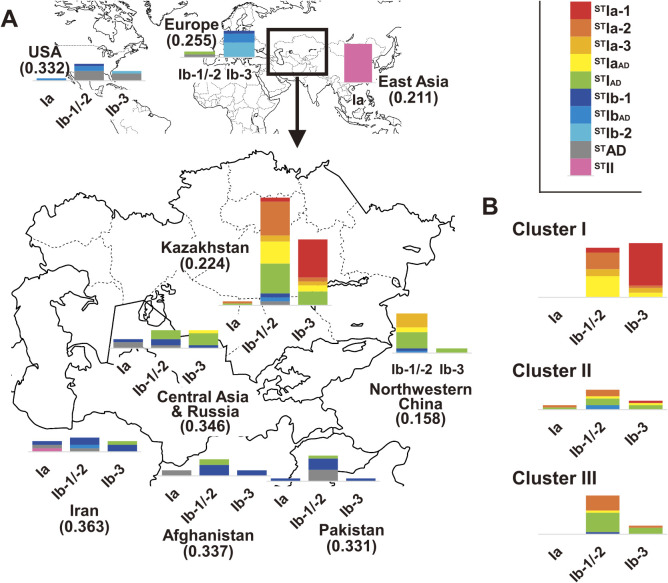
Geographical distribution of melon cytoplasm types and genetic groups after the distance-based method and model-based clustering method. A) Classification of eight areal melons by cytoplasm type and model-based genetic group. Gene diversity is indicated in parentheses. B) Classification of three distance-based genetic groups of Kazakh melon accessions. Bar graph in A) and B) indicates the number of accessions classified into cytoplasm type.

**Fig. 4.**
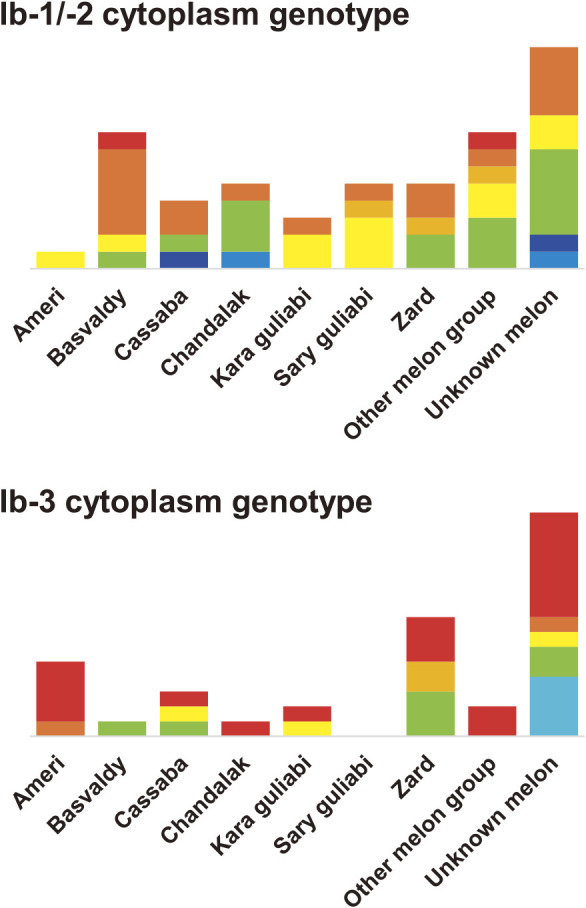
Classification of two cytoplasm types in Kazakh melon groups by the model-based clustering method. Bar indicates the number of accessions classified. The color legend is the same as in Fig. 3.

To visualize the genetic groups associated with Kazakh melon development, the cytoplasm type representative of the maternal lineage was combined with the subgroups from model-based classification and distance-based classification (Fig. 3). Trends from the cytoplasm type were same to those from model-based classification as follows: divergence of East Asian melons from European and American melons and a close relationship between Kazakh melons and those from nearby areas in Central Asia and Russia (Fig. 3A). ^ST^Ib-1 melons and/or Ia cytoplasm melons were rare in Kazakh melons (two accessions). In contrast, in the Ib cytoplasm melons, the subgroup ^ST^IAD of the admixed group with ^ST^Ia and ^ST^Ib was frequent in Central Asian (Turkmenistan, Uzbekistan, and Tajikistan), Russian, northwestern Chinese, and Kazakh melons and thought to be a key genetic group for melon development in these areas. In combination with Ib cytoplasm type, ^ST^Ia-1 with Ib-3 cytoplasm and ^ST^Ia-2 with Ib-1/-2 cytoplasm of Kazakh melons were unique genetic groups in Central Asia. ^ST^Ia-1 with Ib-3 cytoplasm was frequent in Cluster I on UPGMA tree and rare in Cluster II (χ^2^ = 279.4, P < 0.01), whereas ^ST^Ia-2 with Ib-1/-2 cytoplasm was evenly spread across Cluster I to Cluster III (χ^2^ = 5.4, P = 0.25; Fig. 3B). Similar to ^ST^IAD, these two subgroups were detected in the Kazakh melon groups Ameri, Cantalupensis, Chandalak, Zard and Cassaba and subgroups Basvaldy, Guliabi, and Kara Guliabi, as well as in the unknown melon group, and were prevalent in Kazakh melon (Fig. 4). Thus, the three subgroups were indicative of a close relationship between Kazakh melon groups.

### Genetic relationships of Kazakh melon populations with overseas populations

**Fig. 5.**
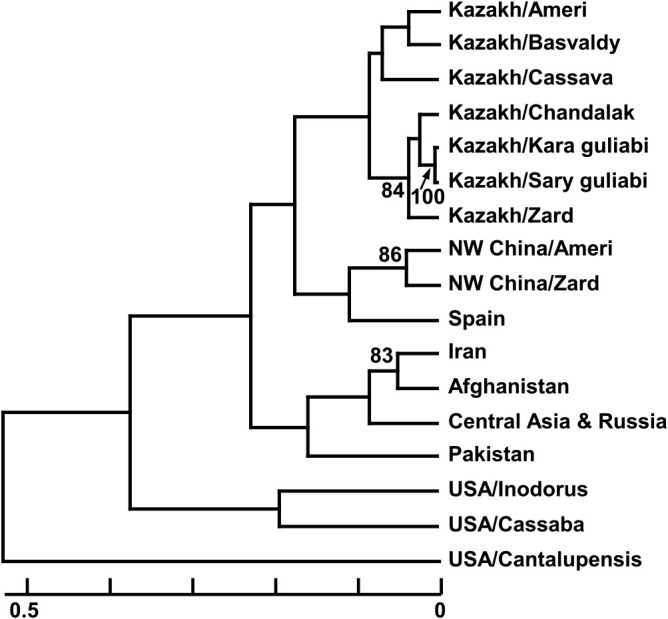
Genetic relationships determined by the UPGMA method based on the genetic distance between 17 melon populations. Bootstrap values over 80% for 1000 replicates are shown beside the tree branch. NW China: Northwestern China.

Close relationship among Kazakh melon groups was supported by the phylogenetic analysis of 17 melon populations including seven Kazakh melon groups and seven overseas populations and their pairwise fixation index (F_ST_) values. Kazakh melon groups clustered on the UPGMA tree, although groups Ameri, Cassaba, and Zard had fruit phenotype similar to the northwestern Chinese Group Ameri, Spanish Group Cassaba, and northwestern Chinese Group Zard, respectively (Fig. 5). This clustering was in agreement with the pairwise F_ST_ values between the 17 melon populations as follows: the pairwise F_ST_ values between six Kazakh melon groups was 0.146 ± 0.053 (range: 0.053–0.236) and were a bit smaller than 0.206 ± 0.074 (range: 0.073–0.406) which was calculated between Kazakh melon groups and populations from nearby Kazakhstan, northwestern China, Iran, Afghanistan, Pakistan, Central Asia including Turkmenistan, Uzbekistan, and Tajikistan and Russia (Table 2). Thus, Kazakh melon groups had genetic similarity with each other, which indicates their low genetic diversity. This low genetic diversity was well supported by the mean gene diversity, being 0.224 for Kazakh melons (Fig. 3A). The values for Kazakh melons were lower than those for Iranian, Afghanistan, Pakistan, and Central Asian and Russian melons (0.331 to 0.363). Thus, the Kazakh melons examined here showed lower genetic variation than other melons. Even between the three Kazakh provincial melon populations from Zhambyl, South Kazakhstan, and Kyzylorda, low divergence was detected by AMOVA, where 3% of the total variance was generated among the populations (Supplemental Fig. 3). The F_ST_ values among these three provincial melon populations was less than 0.036, indicating similar genetic components among them.

## Discussion

Melons are cultivated in southern Kazakhstan and exported to or imported from nearby areas, such as Kyrgyzstan, Turkmenistan, Tajikistan, and Uzbekistan, along the Silk Road. DNA genotyping in Kazakh melon is expected to provide an overview of phylogenetic relationship, diversity, and current conditions of on-farm conservation in these areas. Here, we discuss Kazakh melon development and gene diversity, by taking current melon on-farm conservation into account.

### Kazakh melon development

Model-based classification showed a correlation with distance-based classification and even cytoplasm type. ^ST^IAD with Ib-1/-2 and Ib-3 cytoplasm was frequent in Kazakhstan, Central Asia (Turkmenistan, Uzbekistan, and Tajikistan), Russia, and northwestern China (Fig. 3A). These results support the suggestion of an ancestral relationship among melons along the Silk Road (Aierken *et al.* 2011, Luan *et al.* 2008, Zhang *et al.* 2017) and agree with historical records that Kazakh melon has been inbred in the constituent countries of the former Russian Federation in the Soviet Union era, similar to Kazakh wheat and barley (Almerekova *et al.* 2021, Lister *et al.* 2018, Turuspekov *et al.* 2017). Thus, Kazakh melon is thought to be a progenitor of melons produced through the Silk Road and/or during the Soviet Union era.

From their phylogenetic relationship with ^ST^Ib melons and ^ST^Ia melons (Fig. 1), ^ST^IAD melons appear to be a contributor to Kazakh melon development and are related to both ^ST^Ib-1 melons and ^ST^Ia melons. Two genetic groups, ^ST^Ia-1 with Ib-3 cytoplasm and ^ST^Ia-2 with Ib-1/-2 cytoplasm, were frequent and unique in Kazakh melons (Fig. 3), suggesting their involvement in early Kazakh melon development. The dominance of these two genetic groups in the phylogenetic cluster suggested that the evolution of ^ST^Ia-2 with Ib-1/-2 cytoplasm was prevalent in Clusters I to III, prior to the emergence of ^ST^Ia-1 with Ib-3 cytoplasm (Fig. 3B). However, ^ST^Ia-1 and ^ST^Ia-2 Kazakh melons shared alleles with other Kazakh melons, as well as reference accessions. Frequent recombination events in the whole melon genome are demonstrated by low linkage disequilibrium within diverse melons (Esteras *et al.* 2013, Gonzalo *et al.* 2019). ^ST^Ia-2 and ^ST^Ia-1 Kazakh melons may have been generated by allelic exchange and chromosome recombination.

### What does the genetic variation in Kazakh melon indicate?

Model-based classification suggests that the genetic variation in Kazakh melons formed one admixed group ^ST^IAD and two unique genetic groups: ^ST^Ia-1 with Ib-3 cytoplasm and ^ST^Ia-2 with Ib-1/-2 cytoplasm. A wide variety of Kazakh melons were classified into these three genetic groups (Fig. 3) and showed low mean gene diversity values, similar to northwestern Chinese melons (Fig. 3A). The geographical frequency of ^ST^IAD increased in Central Asia, Kazakhstan, and northwestern China along the eastern Silk Road, compared with those in Iran, Afghanistan, and Pakistan. These results are supported by the study of Aierken *et al.* (2011), who found that northwestern Chinese melons showed less genetic variation than those of Iran, Afghanistan, Pakistan, and Central Asia and Russia, suggesting small size of founder population during Kazakh melon development. In the case of barley, central and northern populations showed genetic distinction from the southern population based on SNPs tightly linked to genes for plant adaptation traits, such as heading date and plant height (Almerekova *et al.* 2021). However, Zhambyl, South Kazakhstan, and Kyzylorda Provinces in southern Kazakhstan, where we collected our plant materials, share suitable conditions for sweet melon production such as low air humidity, large difference in day-and-night air temperature, strong sunshine, and long sunshine duration; these conditions are also found in Uzbekistan and northwestern Chinese areas along the Silk Road (Aierken *et al.* 2011, Mavlyanova *et al.* 2005, Zhang *et al.* 2017). Due to these advantageous conditions for sweet melon cultivation, the selective pressure on melon was weaker and could not drive the decrease in gene diversity in Kazakh melon. Therefore, there are other causes leading to low gene diversity in Kazakh melon, such as genetic drift when melon was introduced into Kazakhstan.

The low genetic variation in Kazakh melon was also explained by the observed heterozygosity, the basal value for the gene diversity. The mean Ho value ranged from 0 to 0.213 in Kazakh melon accessions, which was lower than the He value (range: 0.118–0.574) (Table 1). This reduction in Ho has also been found for Greek and Cypriot melons (Staub *et al.* 2004, Tzitzikas *et al.* 2009), Iranian melons (Raghami *et al.* 2014), and northwestern Chinese melons (Zhang *et al.* 2017). These previous studies suggest the potential for small sample sizes, high levels of selection, or homozygosity by self-pollination causing the reduction in Ho and the low genetic variation in Kazakh melon. Thus, we discuss this potential scenario further.

The current study utilized 89 accessions (one plant each), which generated a mean Ho value of 0.055, or that of 0.06 by rounding the former value off to two decimal places (Table 1). The latter value is lower than that of 0.12 for 360 Iranian melon plants (15 plants each from 24 accessions in Raghami *et al.* 2014), 0.22 for 175 northwestern Chinese melon plants (5 plants each from 35 accessions in Zhang *et al.* 2017), and 0.23 for 500 Indian melon plants (10 plants each from 50 accessions in Fergany *et al.* 2011). The smaller sample size examined in the current study might be prone to the low Ho value, but the Kazakh melon production system has the potential to increase Ho in the future. Different melon groups are cultivated alongside Kazakh melon fields, and seeds for the next round of melon cultivation are harvested from open-pollinated fruits. Cross hybridization between different plants could generate allelic heterozygosity. Andromonoecious plants, i.e., hermaphroditic and male flowers produced on the same individual, were obtained from progenies of the Kazakh melons examined, with the exception of three monoecious accessions (JP242177, JP242179, and JP242183), based on the database from the Genetic Resource Center, NARO, JAPAN (https://www.gene.affrc.go.jp/databases_en.php?section=plant). This sexual type has advantages for pollination, especially self-pollination, because it provides abundant amounts of fertile pollen from the anthers of both hermaphroditic and male flowers during insect-mediated pollination, with equal pollen fertilizing abilities (Fujishita 1959). Thus, andromonoecy leading to self-pollination may promote a decrease in allelic heterozygosity in Kazakh melon.

Close relationships were observed in seven Kazakh melon groups with low F_ST_ values, 0.053 to 0.236 (Table 2); nevertheless, there were specific traits that could be used to classify those seven melon groups (Supplemental Table 2). Several phylogenetic studies display grouping for melons from geographically identical origins but from different horticultural groups, market classes, or cultivar classes (Esteras *et al.* 2013, Raghami *et al.* 2014, Tzitzikas *et al.* 2009), suggesting that the grouping is associated with beneficial traits introgressed into melons and heterogeneity in the examined genomic regions, with retention of potentially important genetic variation. Due to the low genetic variation in Kazakh melon groups, the above patterns suggested that conscious preservation may be performed on specific traits, leading to the retention of their relevant genomic regions, even though other genomic regions may have changed and show genetic similarity within each Kazakh melon group, such as those outside of the genomic region of domesticated genes (Liu *et al.* 2020, Zhao *et al.* 2019).

Several fruit phenotypes were retained in Kazakh melon groups (Supplemental Table 2). Pulp sweetness with less sourness is an essential trait for eating the flesh pulp of cultivated Kazakh melons. Most of the cultivated Kazakh melons had more than 9.0 °Brix (data not shown), which is a normally acceptable value for sweetness in melons (Burger *et al.* 2006). Thick fruit epicarp and hard fruit rind are important shipping-related traits in Kazakh melons, based on the interviews with sellers when Kazakh melon samples were collected. The grade for fruit weight, fruit shape, and fruit length was recognized in different melon groups (Supplemental Fig. 1). Other fruit appearance traits, such as wrinkles, ribs and/or sutures on the fruit surface as well as epicarp color, were easily discriminable traits discriminated for the different melon groups. Fruit pulp color and texture, such as crispy texture and melting texture, might be favorable traits for consumer preference. These fruit trait retained in Kazakh melon may indicate conscious selection by farmers to preserve them to satisfy consumer demands. This preservation may be performed on a melon fruit with traits specific to the melon group that is used for harvesting the seeds for next cultivation, leading to low heterozygosity in the Kazakh melon population (Table 1).

Three melon populations derived from Zhambyl, South Kazakhstan, and Kyzylorda Provinces, the main melon production areas in Kazakhstan, showed low divergence, with an F_ST_ value of less than 0.036. This F_ST_ value was lower than the lowest F_ST_ value of 0.053 among six Kazakh melon groups (Table 2), which appeared to have a similar genetic composition among the three provincial melon populations. Kazakh melons are transported from distant production areas to markets where several melon groups are supplied. Thus, this low divergence reflects the dominant melons in these three provinces and implies that our plant material covers the genetic variation in Kazakh melon.

### Conclusion

The current study provides an overview of Kazakh melon diversity. Distance-based and model-based classifications displayed genetic relationships between Kazakh melon and nearby areal melons and indicated that small populations contributed to melon development and genetic variation in the eastern Silk Road. Close relationships among Kazakh melon groups implied their genetic component resemblance; nevertheless fruit traits specific to the current Kazakh melon groups were conserved, indicating conscious preservation of fruit traits. Information about the diversity and coverage of genetic variation in Kazakh melons is useful for their management and utilization.

## Author Contribution Statement

K.T., R.I., H.N., and K.K. designed the experiments; K.T. and M.S. measured fruit and seed traits; K.T. and Z.M. conducted melon group classification; K.T., G.S., R.M., T.T.D., and Y.A. performed DNA analyses; K.T. and G.S. conducted DNA data analysis; K.T., A.M.A., Z.M. and K.K. interpreted the data; K.T. and K.K. wrote the manuscript; and H.N. and K.K. modified the manuscript. All authors read the manuscript.

## Supplementary Material

Supplemental Figures

Supplemental Tables

## Figures and Tables

**Table 1.** Statistical analysis of genetic variation in RAPD and SSR markers for Kazakh and reference melon accessions

Marker name	Kazakhstan (N = 89)					Reference (N = 113)			
	Na*^a^*	Ne	Ho	He		Na	Ne	Ho	He
RAPD									
A07-872	1	1.000	–	0		2	1.594	–	0.373
A20-1100	2	1.758	–	0.431		2	1.299	–	0.230
A20-800	2	1.046	–	0.044		2	1.366	–	0.268
A22-1520	1	1.000	–	0		2	1.366	–	0.268
A23-1200	2	1.222	–	0.182		2	1.956	–	0.489
A26-1400	2	1.023	–	0.022		2	1.767	–	0.434
A39-2027	2	1.875	–	0.467		2	1.999	–	0.500
A41-930	2	1.169	–	0.145		2	1.213	–	0.176
A57-800	2	1.094	–	0.086		2	1.787	–	0.440
B15-600	2	1.119	–	0.106		2	1.662	–	0.398
B68-1078	2	1.119	–	0.106		2	1.806	–	0.446
B71-1220	2	1.046	–	0.044		2	1.965	–	0.491
B84-700	2	1.144	–	0.126		2	2.000	–	0.500
B84-600	2	1.119	–	0.106		2	1.999	–	0.500
B84-550	2	1.650	–	0.394		2	1.787	–	0.440
B86-1350	1	1.000	–	0		2	1.055	–	0.052
B96-850	2	1.144	–	0.126		2	1.787	–	0.440
B96-750	2	1.277	–	0.217		2	1.965	–	0.491
B99-1400	2	1.046	–	0.044		2	1.639	–	0.390
C00-1350	2	1.023	–	0.022		2	1.503	–	0.335
SSR									
BR12	4	2.398	0.124	0.583		4	2.817	0	0.645
BR22	3	1.741	0.067	0.426		6	3.528	0	0.717
BR53	4	2.729	0.213	0.634		4	2.428	0	0.588
BR83	3	1.494	0	0.331		5	2.482	0	0.597
BR120	2	1.209	0.056	0.173		5	2.353	0	0.575
CMN4-07	4	2.146	0	0.534		7	4.224	0	0.763
CMN4-40	2	1.133	0.011	0.118		6	2.305	0	0.566
CMN08-22	2	1.157	0.011	0.135		2	1.726	0	0.421
CMN08-90	4	1.994	0.079	0.499		4	1.559	0	0.359
CMN21-41	3	1.556	0	0.358		4	2.223	0	0.550
CMN22-16	2	1.998	0.045	0.499		5	2.500	0	0.600

*^a^* Na = Number of alleles, Ne = Number of effective alleles, Ho = Observed heterozygosity, He = Expected heterozygosity.

**Table 2.** Pairwise fixation index (F_ST_) value among 17 melon populations from Kazakhstan and overseas

Country/Melon Group/Province	Kazakhstan								Chinese Hami		Iran	Afghanistan	Pakistan	Central Asia & Russia	Spain	USA		
	Ameri	Basvaldy	Cassaba	Chandalak	Kara guliabi	Sary guliabi	Zard		Ameri	Zard						Honeydew	Cassaba	Cantalupensis
Kazakhstan/Ameri																		
/Basvaldy	0.145*^a^																	
/Cassaba	0.091	0.090																
/Chandalak	0.195**	0.164*	0.108															
/Kara guliabi	0.201*	0.208**	0.159*	0.133														
/Sary guliabi	0.236**	0.201**	0.160*	0.175**	0.226*													
/Zard	0.099*	0.111***	0.062	0.053	0.121*	0.115*												
Northwestern China/Ameri	0.205***	0.224***	0.152**	0.179***	0.258***	0.243***	0.114***											
/Zard	0.330**	0.337**	0.233**	0.283**	0.406**	0.361**	0.179***		0.080									
Iran	0.242***	0.220***	0.139**	0.199***	0.289***	0.273***	0.160***		0.184***	0.243**								
Afghanistan	0.193***	0.195***	0.105**	0.188***	0.243***	0.233**	0.136***		0.171***	0.222**	0.064							
Pakistan	0.214***	0.195***	0.129**	0.226***	0.263**	0.258***	0.177***		0.194***	0.268***	0.100***	0.093**						
Central Asia*^b^* & Russia	0.141*	0.134***	0.074	0.113*	0.174**	0.163**	0.073**		0.114***	0.176**	0.077*	0.063	0.095**					
Spain	0.337***	0.303***	0.206**	0.255***	0.358***	0.362**	0.251***		0.211***	0.351***	0.190***	0.194***	0.229***	0.160***				
USA/Honeydew	0.649**	0.557***	0.414**	0.470**	0.644**	0.657**	0.442***		0.446**	0.584*	0.344***	0.388**	0.431**	0.286***	0.408**			
/Cassaba	0.404**	0.355**	0.234*	0.319**	0.432**	0.418*	0.294***		0.304***	0.394*	0.155*	0.184**	0.221***	0.153*	0.223**	0.349*		
/Cantalupensis	0.392***	0.333***	0.268***	0.337**	0.413***	0.392**	0.296***		0.346***	0.418**	0.187***	0.188***	0.174***	0.139***	0.347***	0.507**	0.323**	

*^a^* Significance at the * 5%, ** 1% level, and *** 0.1% level. *^b^* Turkmenistan, Uzbekistan, and Tajikistan.
