# Supplementary material for: Melon diversity on the Silk Road by molecular phylogenetic analysis in Kazakhstan melons
Source: Breed Sci. 2023 Apr 25;73(2):219–29. doi: 10.1270/jsbbs.22030 (PMC10316308; doi:10.1270/jsbbs.22030)
Supplement: Supplementary file 1 — Supplemental Figures [file 73_219_s1.pdf]

doi: 10.1270/jsbbs.22030

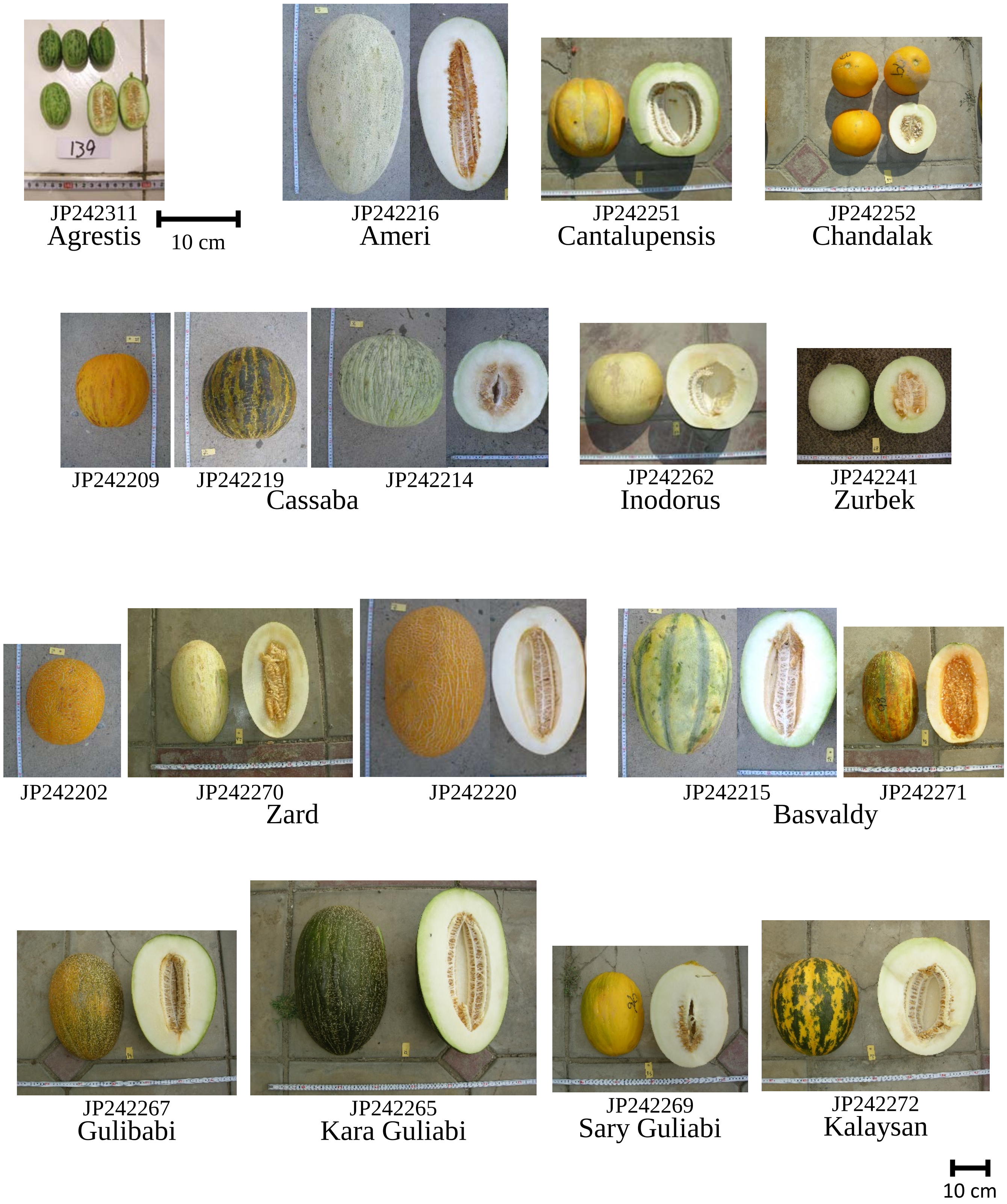

Supplemental Fig. 1 Representative melon fruits of seven groups and six subgroups of Group Zard in Kazakhstan.

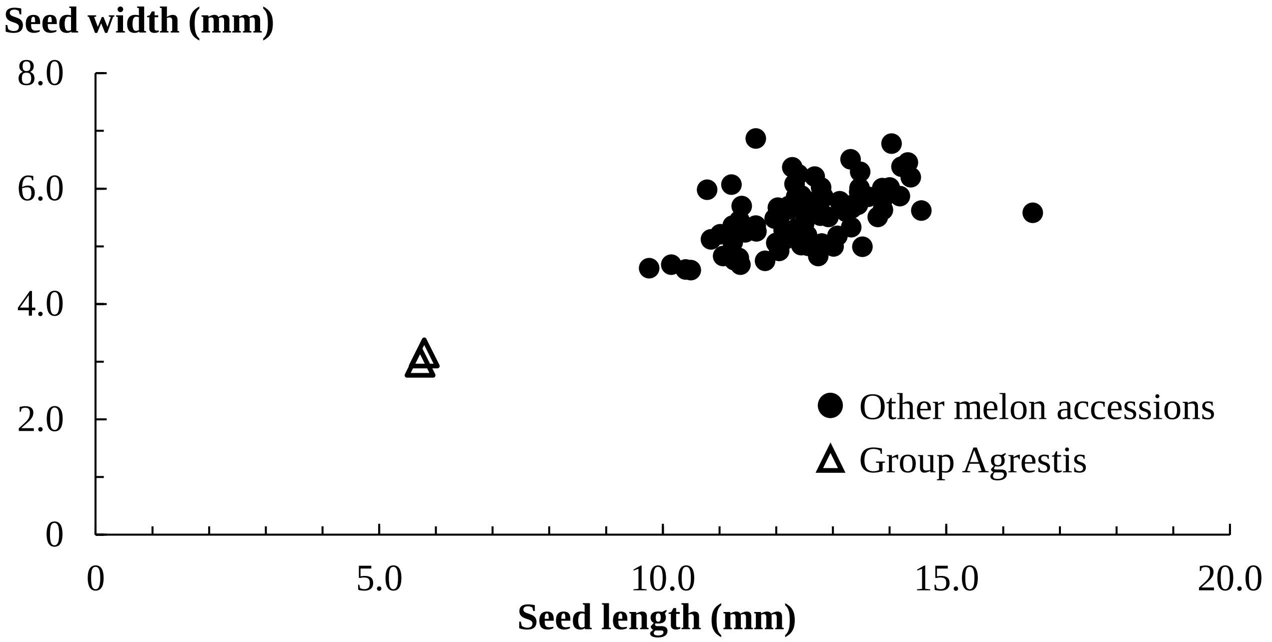

Supplemental Fig. 2 Variations of seed length and seed width in 87 Kazakh melon accessions.

A

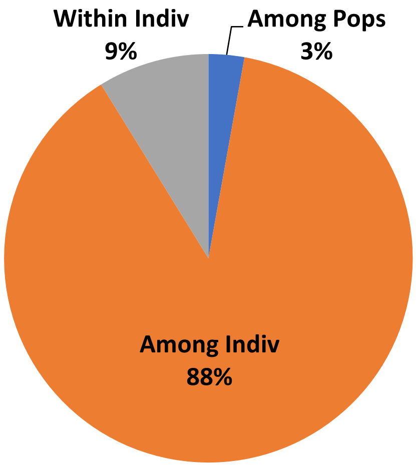

Supplemental Fig. 3 Analysis of molecular variance between three Kazakh areal melon populations.
