# Supplementary material for: Melon diversity on the Silk Road by molecular phylogenetic analysis in Kazakhstan melons
Source: Breed Sci. 2023 Apr 25;73(2):219–29. doi: 10.1270/jsbbs.22030 (PMC10316308; doi:10.1270/jsbbs.22030)
Supplement: Supplementary file 2 — Supplemental Tables [file 73_219_s2.pdf]

Supplemental Table 1 Details of melon accessions analysed in this study

Accession number	Cultivar name[a]	Seed source[b]	Group	Sub-Group	Country of origin/ Province/Site name	Seed type	Cytoplasm type	Cluster no. in UPGMA	Group after STRUCTURE
Kazakh melon accession									
JP242177	KSS001	1	Zard	Zard	Kazakhstan/Almaty	Large	Ia	II	Ia-2
JP242178	KSS002	1	Chandalak		Kazakhstan/Almaty	Large	Ib-1/-2	II	IAD
JP242179	KSS005	1	Unknown		Kazakhstan/Zhambyl	Large	Ib-3	II	IAD
JP242180	KSS006	1	Zard	Zard	Kazakhstan/Zhambyl	Large	Ib-3	II	IAD
JP242181	KSS007	1	Zard	Zard	Kazakhstan/Zhambyl	Large	Ib-1/-2	V	IAD
JP242182	KSS008	1	Chandalak		Kazakhstan/Zhambyl	Large	Ib-1/-2	II	IAD
JP242183	KSS009	1	Zard	Zard	Kazakhstan/Zhambyl	Large	Ib-3	I	Ia-1
JP242184	KSS010	1	Unknown		Kazakhstan/Zhambyl	Large	Ib-3	I	Ia-1
JP242187	KSS013	1	Zard	Zard	Kazakhstan/Zhambyl	Large	Ib-1/-2	I	Ia-2
JP242188	KSS014	1	Unknown group		Kazakhstan/Zhambyl	Large	Ib-3	III	IAD
JP242189	KSS015	1	Chandalak		Kazakhstan/Zhambyl	Large	Ib-1/-2	I	Ia-2
JP242190	KSS016	1	Zard	Guliabi	Kazakhstan/Zhambyl	Large	Ib-3	I	Ia-1
JP242191	KSS017	1	Zard	Zard	Kazakhstan/Zhambyl	Large	Ib-1/-2	I	Ia-3
JP242192	KSS018	1	Unknown		Kazakhstan/Zhambyl	Large	Ib-1/-2	I	Ia-2
JP242193	KSS019	1	Zard	Zard	Kazakhstan/Zhambyl	Large	Ib-1/-2	II	Ia-2
JP242194	KSS020	1	Zard	Zard	Kazakhstan/Zhambyl	Large	Ib-3	I	Ia-1
JP242195	KSS021	1	Unknown group		Kazakhstan/Zhambyl	Large	Ib-1/-2	I	Ia-2
JP242196	KSS022	1	Zard	Basvaldy	Kazakhstan/Zhambyl	Large	Ib-1/-2	III	Ia-2
JP242197	KSS023	1	Unknown		Kazakhstan/Zhambyl	Large	Ib-1/-2	III	IAD
JP242198	KSS024	1	Unknown		Kazakhstan/Zhambyl	Large	Ib-1/-2	III	Ia-2
JP242200	KSS026	1	Zard	Zard	Kazakhstan/Zhambyl	Large	Ib-3	I	Ia-1
JP242201	KSS027	1	Unknown group		Kazakhstan/Zhambyl	Large	Ib-3	III	Ia-2
JP242202	KSS028	1	Zard	Zard	Kazakhstan/Zhambyl	Large	Ib-1/-2	IV	IAD
JP242203	KSS029	1	Zard	Sary guliabi	Kazakhstan/Zhambyl	Large	Ib-1/-2	III	Ia-AD
JP242204	KSS030	1	Chandalak		Kazakhstan/Zhambyl	Large	Ib-3	I	Ia-1
JP242205	KSS031	1	Unknown group		Kazakhstan/Zhambyl	Large	Ib-3	I	Ia-1
JP242206	KSS032	1	Cantalupensis		Kazakhstan/Zhambyl	Large	Ib-1/-2	III	Ia-2
JP242207	KSS033	1	Zard	Basvaldy	Kazakhstan/Zhambyl	Large	Ib-1/-2	I	Ia-1
JP242208	KSS034	1	Cantalupensis		Kazakhstan/Zhambyl	Large	Ib-1/-2	I	Ia-1
JP242209	KSS035	1	Cassaba		Kazakhstan/Zhambyl	Large	Ib-3	II	Ia-1
JP242210	KSS036	1	Zard	Basvaldy	Kazakhstan/Zhambyl	Large	Ib-3	III	IAD
JP242211	KSS037	1	Cassaba		Kazakhstan/Zhambyl	Large	Ib-1/-2	II	Ia-2
JP242213	KSS039	1	Zard	Basvaldy	Kazakhstan/Zhambyl	Large	Ib-1/-2	III	Ia-2
JP242214	KSS040	1	Cassaba		Kazakhstan/South Kazakhstan	Large	Ib-1/-2	III	IAD
JP242215	KSS041	1	Zard	Basvaldy	Kazakhstan/South Kazakhstan	Large	Ib-1/-2	I	Ia-AD
JP242216	KSS043	1	Ameri		Kazakhstan/South Kazakhstan	Large	Ib-3	I	Ia-1
JP242217	KSS044	1	Ameri		Kazakhstan/South Kazakhstan	Large	Ib-3	I	Ia-2
JP242218	KSS045	1	Unknown group		Kazakhstan/South Kazakhstan	Large	Ib-1/-2	III	Ia-2
JP242219	KSS046	1	Cassaba		Kazakhstan/South Kazakhstan	Large	Ib-3	I	Ia-AD
JP242220	KSS047	1	Zard	Zard	Kazakhstan/South Kazakhstan	Large	Ib-3	I	Ia-3
JP242221	KSS048	1	Unknown group		Kazakhstan/South Kazakhstan	Large	Ib-3	I	Ia-1
JP242222	KSS049	1	Zard	Zard	Kazakhstan/South Kazakhstan	Large	Ib-3	I	Ia-3
JP242223	KSS050	1	Zard	Zard	Kazakhstan/South Kazakhstan	Large	Ib-3	III	IAD
JP242225	KSS052	1	Zard	Basvaldy	Kazakhstan/Kyzylorda	Large	Ib-1/-2	III	Ia-2
JP242226	KSS053	1	Unknown group		Kazakhstan/Kyzylorda	Large	Ib-3	I	Ia-1
JP242227	KSS054	1	Cassaba		Kazakhstan/Kyzylorda	Large	Ib-3	V	IAD
JP242228	KSS055	1	Zard	Kara guliabi	Kazakhstan/Kyzylorda	Large	Ib-1/-2	I	Ia-AD
JP242229	KSS056	1	Zard	Sary guliabi	Kazakhstan/Kyzylorda	Large	Ib-1/-2	I	Ia-AD
JP242230	KSS057	1	Unknown		Kazakhstan/Kyzylorda	Large	Ib-1/-2	I	Ia-AD
JP242231	KSS058	1	Cassaba		Kazakhstan/Kyzylorda	Large	Ib-1/-2	III	Ib-1
JP242233	KSS060	1	Unknown		Kazakhstan/Kyzylorda	Large	Ib-3	I	Ia-1
JP242234	KSS061	1	Zurbek		Kazakhstan/Kyzylorda	Large	Ib-1/-2	I	Ia-AD
JP242235	KSS062	1	Ameri		Kazakhstan/Kyzylorda	Large	Ib-3	I	Ia-1
JP242236	KSS063	1	Zard	Zard	Kazakhstan/Kyzylorda	Large	Ib-3	V	IAD
JP242237	KSS064	1	Unknown group		Kazakhstan/Kyzylorda	Large	Ib-3	I	Ia-1
JP242238	KSS065	1	Zard	Sary guliabi	Kazakhstan/Kyzylorda	Large	Ib-1/-2	I	Ia-3
JP242239	KSS066	1	Cassaba		Kazakhstan/Kyzylorda	Large	Ib-1/-2	I	Ia-2
JP242240	KSS067	1	Cantalupensis		Kazakhstan/Kyzylorda	Large	Ib-1/-2	III	IAD
JP242241	KSS068	1	Zurbek	Zurbek	Kazakhstan/Kyzylorda	Large	Ib-1/-2	IV	IAD
JP242242	KSS069	1	Zard	Kara guliabi	Kazakhstan/Kyzylorda	Large	Ib-3	I	Ia-1
JP242244	KSS071	1	Ameri		Kazakhstan/Kyzylorda	Large	Ib-3	I	Ia-1
JP242245	KSS072	1	Unknown group		Kazakhstan/Kyzylorda	Large	Ib-1/-2	III	IAD
JP242246	KSS073	1	Unknown group		Kazakhstan/Kyzylorda	Large	Ib-3	I	Ia-AD
JP242247	KSS074	1	Unknown group		Kazakhstan/Kyzylorda	Large	Ib-3	I	Ia-1
JP242248	KSS075	1	Ameri		Kazakhstan/Kyzylorda	Large	Ib-3	I	Ia-1
JP242249	KSS076	1	Zard	Kara guliabi	Kazakhstan/Kyzylorda	Large	Ib-3	II	Ia-AD
JP242251	KSS078	1	Unknown group		Kazakhstan/Kyzylorda	Large	Ib-1/-2	III	IAD
JP242252	KSS079	1	Chandalak		Kazakhstan/Kyzylorda	Large	Ib-1/-2	III	IAD
JP242253	KSS080	1	Zard	Basvaldy	Kazakhstan/Kyzylorda	Large	Ib-1/-2	I	Ia-2
JP242254	KSS081	1	Zard	Kara guliabi	Kazakhstan/Kyzylorda	Large	Ib-1/-2	II	Ia-2
JP242255	KSS082	1	Zard	Sary guliabi	Kazakhstan/Kyzylorda	Large	Ib-1/-2	III	Ia-2
JP242256	KSS083	1	Zard	Basvaldy	Kazakhstan/Kyzylorda	Large	Ib-1/-2	III	IAD
JP242257	KSS084	1	Unknown		Kazakhstan/Kyzylorda	Large	Ib-1/-2	IV	Ib-1
JP242260	KSS087	1	Unknown group		Kazakhstan/Kyzylorda	Large	Ib-1/-2	II	Ia-AD
JP242261	KSS088	1	Unknown group		Kazakhstan/Kyzylorda	Large	Ib-1/-2	II	IAD
JP242262	KSS089	1	Inodorus		Kazakhstan/Kyzylorda	Large	Ib-1/-2	III	IAD
JP242263	KSS090	1	Unknown		Kazakhstan/Kyzylorda	Large	Ib-1/-2	III	IAD
JP242264	KSS091	1	Kalaysan		Kazakhstan/Kyzylorda	Large	Ib-1/-2	I	Ia-AD
JP242265	KSS092	1	Zard	Kara guliabi	Kazakhstan/Kyzylorda	Large	Ib-1/-2	I	Ia-AD
JP242266	KSS093	1	Ameri		Kazakhstan/Kyzylorda	Large	Ib-1/-2	I	Ia-AD
JP242267	KSS094	1	Zard	Guliab	Kazakhstan/Kyzylorda	Large	Ib-3	I	Ia-1
JP242269	KSS096	1	Zard	Sary guliabi	Kazakhstan/Kyzylorda	Large	Ib-1/-2	I	Ia-AD
JP242270	KSS097	1	Zard	Zard	Kazakhstan/Kyzylorda	Large	Ia	II	IAD
JP242271	KSS098	1	Zard	Basvaldy	Kazakhstan/Kyzylorda	Large	Ib-1/-2	I	Ia-2
JP242272	KSS099	1	Kalaysan		Kazakhstan/Kyzylorda	Large	Ib-1/-2	I	Ia-3
JP242310	KSS138	1	Agrestis		Kazakhstan/Kyzylorda	Small	Ib-1/-2	V	AD
JP242311	KSS139	1	Agrestis		Kazakhstan/Kyzylorda	Small	Ib-1/-2	V	AD

Supplemental Table 1 (continued)

Accession number	Cultivar name[a]	Seed source[b]	Group	Sub-Group	Country of origin/ Province/Site name	Seed type	Cytoplasm type	Cluster no. in UPGMA	Group after STRUCTURE
Reference accession									
JP32114	Honey Dew	2	Inodorus	Honeydew	USA	Large	Ib-3	IV	Ib-2
JP138196	Honey Dew	2	Inodorus	Honeydew	USA	Large	Ib-3	IV	Ib-2
JP138219	Honey Dew	2	Inodorus	Honeydew	USA	Large	Ib-3	IV	Ib-2
JP138302	Honey Dew	2	Inodorus	Honeydew	USA	Large	Ib-3	IV	Ib-2
NSL 20616	FLORIDEW	3	Cassaba		USA	Large	Ib-3	IV	Ib-2
NSL 5647	GOLDEN CRENSHAW	3	Cassaba		USA	Large	Ib-1/-2	IV	Ib-AD
NSL 5659	GOLDEN BEAUTY CASABA	3	Cassaba		USA	Large	Ib-1/-2	IV	Ib-AD
NSL 5709	SUNGOLD CASABA	3	Cassaba		USA	Large	Ia	IV	Ib-AD
JP77631	Rocky Ford	2	Cantalupensis		USA	Large	Ib-1/-2	VI	AD
JP88366	Homegarden	2	Cantalupensis		USA	Large	Ib-3	VI	AD
JP132711	Georgia 47	2	Cantalupensis		USA	Large	Ib-1/-2	VI	AD
JP138212	♯58-21	2	Cantalupensis		USA	Large	Ib-3	VI	AD
JP83516	SC108 (C-108)	2	Cantalupensis		USA	Large	Ib-1/-2	V	Ib-1
JP83519	Rio Gold	2	Cantalupensis		USA	Large	Ib-1/-2	VI	AD
JP83521	Hales Best	2	Cantalupensis		USA	Large	Ib-1/-2	VI	AD
JP32115	Spicy	2	Cantalupensis		USA	Large	Ib-3	VI	AD
JP216624	Earl's Favourite	2	Inodorus		England	Large	Ib-1/-2	III	IAD
JP132642	Melon Cantalupo[1]	2	Cantalupensis		France	Large	Ib-1/-2	VI	AD
JP77616	Tendral[1]	2	Ibericus		Spain	Large	Ib-3	III	Ib-1
PI 512411	–	3	Unknown		Spain/Zaragoza	Large	Ib-3	IV	Ib-2
PI 512413	–	3	Unknown		Spain/Zaragoza	Large	Ib-3	IV	Ib-2
PI 512462	–	3	Unknown		Spain/Cadiz	Large	Ib-3	IV	Ib-2
PI 512489	–	3	Unknown		Spain/Lerida	Large	Ib-3	IV	Ib-AD
PI 512501	–	3	Unknown		Spain/Caceres	Small,large	Ib-3	IV	Ib-2
PI 512510	–	3	Unknown		Spain/Badajoz	Large	Ib-3	IV	Ib-AD
PI 512564	–	3	Unknown		Spain/Valencia	Large	Ib-3	IV	Ib-AD
PI 512581	–	3	Unknown		Spain/Castellon de Plana	Small,large	Ib-3	IV	Ib-2
PI 140666	–	3	Unknown		Iran/Mazandaran	Large	Ib-1/-2	III	Ib-1
PI 140814	–	3	Unknown		Iran/Khorasan	Large	Ib-3	III	IAD
PI 143231	–	3	Unknown		Iran/West Azerbaijan	Large	Ib-3	III	Ib-1
PI 230185	–	3	Unknown		Iran/Gorgab	Large	Ib-1/-2	VI	AD
PI 137834	–	3	Unknown		Iran/Kerman	Large	Ib-3	V	Ib-1
PI 140675	–	3	Unknown		Iran/Mazandaran	Large	Ib-1/-2	III	Ib-1
PI 211922	–	3	Unknown		Iran	Large	Ia	VII	II
PI 211923	–	3	Unknown		Iran	Small	Ia	V	AD
PI 211942	–	3	Unknown		Iran	Small	Ia	V	Ib-1
PI 351132	–	3	Unknown		Iran	Large	Ib-1/-2	IV	Ib-AD
CUM 254	–	4	Dudaim		Afghanistan	Small,large	Ia	V	AD
PI 125942	–	3	Unknown		Balkh/Afghanistan	Large	Ib-3	V	Ib-1
PI 126050	–	3	Unknown		Badakhshan/Afghanistan	Large	Ib-3	IV	Ib-1
PI 127534	–	3	Unknown		Samangan/Afghanistan	Large	Ib-1/-2	III	Ib-1
PI 212089	–	3	Unknown		Herat/Afghanistan	Large	Ib-1/-2	III	IAD
PI 126090	–	3	Unknown		Takhar/Afghanistan	Small	Ia	VI	AD
PI 126105	–	3	Unknown		Jowzjan/Afghanistan	Small,large	Ib-1/-2	III	Ib-1
PI 127550	–	3	Unknown		Ghazni/Afghanistan	Large	Ib-1/-2	III	IAD
PI 207478	–	3	Unknown		Kabul/Afghanistan	Large	Ib-1/-2	III	Ib-1
PI 220515	–	3	Unknown		Helmand/Afghanistan	Small	Ib-1/-2	V	Ib-1
PI 116824	–	3	Unknown		Punjab/Pakistan	Large	Ib-1/-2	III	IAD
PI 123188	–	3	Unknown		Pakistan	Small	Ib-1/-2	VI	AD
PI 124552	–	3	Unknown		Pakistan/Sind	Large	Ib-1/-2	V	AD
PI 124553	–	3	Unknown		Pakistan/Sind	Large	Ib-1/-2	V	Ib-1
PI 163211	–	3	Unknown		Pakistan/Punjab	Small	Ib-1/-2	V	Ib-1
PI 217525	–	3	Unknown		Pakistan/Punjab	Small	Ib-1/-2	III	Ib-1
PI 217945	–	3	Unknown		Pakistan	Small	Ib-1/-2	V	AD
PI 218070	–	3	Unknown		Pakistan/Punjab	Small	Ib-1/-2	V	Ib-1
PI 218071	–	3	Unknown		Pakistan	Small	Ib-1/-2	V	AD
PI 426629	–	3	Unknown		Pakistan/Mingora	Small	Ia	IV	Ib-1
PI 532929	–	3	Unknown		Pakistan/Skardu	Large	Ib-3	IV	Ib-1
P173	Altajskaja skorospelaja	1	Unknown		Russia/Altai	Large	Ib-1/-2	VI	AD
P174	Barnaulka	1	Unknown		Russia/Altai	Small	Ib-1/-2	III	Ib-1
P175	Kolkhoznitsa	1	Unknown		Russia/Ukrine	Large	Ib-1/-2	III	IAD
PI 476342	Imljskaja/VIR 6809	3	Chandalak		Kazakhstan	Large	Ib-1/-2	II	Ib-AD
Ames 19036	–	3	Unknown		Kazakhstan/Almaty	Large	Ib-1/-2	II	Ib-AD
PI 476331	Zaami 672/VIR 40689	3	Unknown		Turkmenistan	Large	Ib-1/-2	III	IAD
CUM 209	–	4	Agrestis		Turkmenistan	Small	Ia	V	Ib-1
P250	P250	1	Unknown		Turkmenistan	Large	Ia	VI	AD
P251	P251	1	Unknown		Turkmenistan	Small	Ia	VI	AD
PI 476333	Kokca 588/VIR 5149	3	Unknown		Uzbekistan	Large	Ib-3	I	IAD
PI 476337	Sakor-polak 554/VIR 5883	3	Unknown		Uzbekistan	Large	Ib-3	V	Ib-1
JP82398	Kokand	2	Ameri		Uzbekistan	Large	Ib-3	II	IAD
JP82399	Mirzuchulskaja	2	Ameri		Uzbekistan	Large	Ib-3	III	IAD
JP134722	Ak-Urug	2	Ameri		Uzbekistan	Large	Ib-3	I	Ia-AD
CUM 333	Dushanbe	4	Unknown		Tajikistan	Large	Ib-3	III	IAD
CUM 334	Dushanbe	4	Inodorus		Tajikistan	Large	Ib-1/-2	III	IAD
CUM 389	Dushanbe	4	Unknown		Tajikistan	Large	Ib-1/-2	III	Ib-1
X001-3	Laoguniang	1	Ameri		China/Xinjiang	Large	Ib-1/-2	III	Ib-1
X002-2	Mizigua	1	Ameri		China/Xinjiang	Large	Ib-1/-2	IV	IAD
X003-1	Kukeqi	1	Ameri		China/Xinjiang	Large	Ib-1/-2	II	Ia-3
X005-1	Paotaihong	1	Local variety	Zard	China/Xinjiang	Large	Ib-1/-2	II	Ia-3
X006-1	Hongxincui	1	Ameri		China/Xinjiang	Large	Ib-1/-2	II	IAD
X007-1	Wanshudonggua	1	Local variety	Zard	China/Xinjiang	Large	Ib-1/-2	II	IAD
X008-3	Kakeqie	1	Ameri		China/Xinjiang	Large	Ib-1/-2	II	IAD
X009	Bixiekexin	1	Cassaba		China/Xinjiang	Large	Ib-1/-2	II	IAD
X010	Kuche	1	Ameri		China/Xinjiang	Large	Ib-1/-2	II	Ia-3
X011-1	Huanghou	1	Ameri		China/Xinjiang	Large	Ib-3	II	IAD
X012	Bawudong	1	Ameri	Ameri	China/Xinjiang	Large	Ib-1/-2	II	Ib-AD
X013	Xiekesu	1	Ameri	Ameri	China/Xinjiang	Large	Ib-1/-2	I	Ia-AD

Supplemental Table 1 (continued)

Accession number	Cultivar name[a]	Seed source[b]	Group	Sub-Group	Country of origin/ Province/Site name	Seed type	Cytoplasm type	Cluster no. in UPGMA	Group after STRUCTURE
X014	Kashi	1	Ameri		China/Xinjiang	Large	Ib-1/-2	II	Ia-3
X021-1	Baipicui	1	Ameri	Ameri	China/Xinjiang	Large	Ib-1/-2	I	Ia-AD
X023-1	Kalakusai	1	Local variety	Zard	China/Xinjiang	Large	Ib-1/-2	II	IAD
X024-1	Kaernaishi	1	Ameri		China/Xinjiang	Large	Ib-1/-2	II	Ia-3
X030	Wangwenxiang	1	Ameri	Ameri	China/Xinjiang	Large	Ib-1/-2	II	IAD
X031	Heimeimaomijigan	1	Local variety	Zard	China/Xinjiang	Large	Ib-1/-2	II	IAD
X032	Kutuerkukeqi	1	Ameri		China/Xinjiang	Large	Ib-1/-2	II	Ia-3
CYW37	–	1	Unknown		China/Yunnan	Small	Ia	VII	II
CYW38	–	1	Unknown		China/Yunnan	Small	Ia	VII	II
CYW49	–	1	Unknown		China/Yunnan	Small	Ia	VII	II
CYW60	–	1	Unknown		China/Yunnan	Small	Ia	VII	II
CYW61	–	1	Unknown		China/Yunnan	Small	Ia	VII	II
C28	Xingtangmiangua	1	Makuwa		China	Small	Ia	VII	II
JP74157	Mi-tang-tin	2	Makuwa		China	Small	Ia	VII	II
JP138556	Damiangua	2	Makuwa		China	Small	Ia	VII	II
JP138557	Shidaodaqinggua	2	Makuwa		China	Small	Ia	VII	II
JP216363	Shilinghuangjingua	2	Makuwa		China	Small	Ia	VII	II
JP74164	Wengua	2	Makuwa		China	Small	Ia	VII	II
JP88375	Chi-86-56	2	Makuwa		China	Small	Ia	VII	II
JP88380	Qianzhong-5	2	Makuwa		China	Small	Ia	VII	II
JP204561	Chi-87-12	2	Makuwa		China	Small	Ia	VII	II
C32	Heipilengzisudigua	1	Conomon		China	Small	Ia	VII	II
JP88378	Chi-86-61	2	Conomon		China	Small	Ia	VII	II
JP83506	Qingpilürouxianggua	2	Conomon		China	Small	Ia	VII	II
P169	Caigua	2	Conomon		China	Small	Ia	VII	II
P171	Qingpicaigua	2	Conomon		China	Small	Ia	VII	II
JP32119	Kinpyo	2	Makuwa		Japan	Small	Ia	VII	II
JP32205	Karimori	2	Conomon		Japan	Small	Ia	VII	II

[a] Cultivars are indicated by the name registered in the donor institutions. ‘Melon Cantalupo’ = ‘Melon Cantalupo di Charentais’. ‘Tendral’ = ‘Tendral o Invernale a Buccia Verde’.

[b] Seeds were provided by four institutes indicated by the following abbreviations: 1 = Okayama University, Japan; 2 = Institute of Vegetable and Floriculture Science (NIVFS), National Agriculture and Food Research Organization (NARO), Japan; 3 = North Central Regional Plant Introduction Station, Iowa State University (USDA-ARS), USA; and 4 = Leibniz Institute of Plant Genetics and Crop Plant Research (IPK), Germany.

Supplemental Table 2 Fruit trait description for 13 Kazakh melon groups

Group or Subgroup name	Fruit description
Agrestis	Very small fruits (around 50 g); elliptic fruit shape; no ribs, no netted, depthless wrinkled fruit surface with pileous short hairs; fruit light-green uniform color with dark-greens spots; thin exocarp; light-green to white pulp; no cavity; no aromatic fruit; presence of a gelatinous sheath around small seeds.
Ameri	Medium to high fruit weight; oval or cylindrical fruit shape; presence or absence of netting, no ribs, not wrinkled fruit surface; white or light-orange pulp color; sweet pulp with sometimes crispy texture; three plracentas/locules; cavity in the locules; no aromatic fruit; a gelatinous sheath around large seeds.
Cantalupensis	Medium fruit weight; flat or round fruit shape; presence or absence of netting, ribs, not wrinkled fruit surface; orange exocarp color; white to light-green or orange pulp color; sweet pulp with juicy flesh texture; three plracentas/locules; large cavity in the locules; a gelatinous sheath around large seeds.
Cassaba	Small to medium fruit weight; slightly flat to round fruit shape; presence or absence of netting, no ribs, more or less wrinkled fruit surface; white to light-green pulp color; thick, sweet pulp with slight hard texture; three plracentas/locules; less cavity in the locules; no aromatic fruit; a gelatinous sheath around large seeds.
Chandalak	Small fruit weight; flat to round fruit shape; no netting, no rib, not wrinkled fruit surface; yellow or dark yellow epicarp color; white pulp color; sometime thick, sweet pulp; three plracentas/locules; somotimes no cavity in the locules; no aromatic fruit; a gelatinous sheath around large seeds.
Inodorus	Medium fruit weight, round fruit shape; netting, no ribs, not wrinkled fruit surface; light-yellow epicarp color; white pulp color; thick, high sugar content pulp with juicy texture; three plracentas/locules; large cavity in the locules; no aromatic fruit; a gelatinous sheath around large seeds.
Zurbek	Medium fruit weight; round fruit shape; no netting, no ribs, not wrinkled, smooth fruit surface; light-green epicarp color; white to light-green pulp color; thick, sweet pulp with slightly hard texture; three plracentas/locules; less or no cavity in the locules; no aromatic fruit; a gelatinous sheath around large seeds.
Basvaldy	Medium to high fruit weight; oval or elliptic fruit shape; no netting, green or light-green or white colored ribs or sutures, not wrinkled, smooth fruit surface; yellow, orange epicarp color; white to light-green, green, orange pulp color; sweet pulp with slightly weak texture; three plracentas/locules; cavity in the locules; no aromatic fruit; a gelatinous sheath around large seeds.
Guliabi	Medium to high fruit weight; elliptic or acorn fruit shape; presence or less of netting, no ribs, less wrinkled fruit surface; yellow with a partly green epicarp color; white pulp color; very thick, sweet pulp with slightly hard texture; three plracentas/locules; cavity in the locules; no aromatic fruit; a gelatinous sheath around large seeds.
Kalaysan	Medium fruit weight; elliptic or acorn fruit shape; no netting, no ribs, no wrinkled, smooth fruit surface; yellow with green patches colored stripes epicarp color; white pulp color; very thick, sweet pulp with slightly hard texture; three plracentas/locules; sometimes less cavity in the locules; three plracentas; no aromatic fruit; a gelatinous sheath around large seeds.
Kara guliabi	High fruit weight; elliptic or acorn fruit shape; less or no netting, no ribs, wrinkled fruit surface; light-green, green epicarp color; white pulp color; very thick, sweet pulp with slightly hard texture; three plracentas/locules; sometimes less cavity in the locules; no aromatic fruit; a gelatinous sheath around large seeds.
Sary guliabi	Small to medium fruit weight; elliptic or acorn fruit shape; less or no netting, no ribs, wrinkled fruit surface; yellow epicarp color; white pulp color; very thick, sweet pulp with slightly hard texture; three plracentas/locules; sometimes less cavity in the locules; no aromatic fruit; a gelatinous sheath around large seeds.
Zard	Small to high fruit weight; round, oval, elliptic fruit shape; presence or slightly netting, no ribs, no wrinkled fruit surface; yellow or light-green epicarp color; white pulp color; thick (high fruit weight), sweet pulp; three plracentas/locules; cavity in the locules; no aromatic fruit; a gelatinous sheath around large seeds.

Supplemental Table 3 Molecular markers used in this study

InDel, SNP[a] name/Primer name	F primer sequence/[b] RAPD primer sequence (5' → 3')	R primer sequence(5' → 3')	PCR product size/[c] Size of polymorphic fragment (bp)	Position in the[c] chloroplast genome	Restriction enzyme
Chloroplast genome marker					
InDel1	CGGGAAGGGCTCGKGCAG	GTTCGAATCCCTCTCTCTCCTTTT	339/334	37,347	
SNP2	AATATCCAAATACCAAATT**g**T	TCGGAATTATTGGAAGAATTCTT	117	1,974	*Rsa* I
SNP18	AAAAAAAAACAATTGCAGATT**r**A**a**TT	TGCAGCATTTAAAAGGGTCTGAGGT	137	83,140	*Apo* I
SNP19	GTAAAATTTTTTGACAAT**t**TA	AATTATTCTTTCTTGCTCTAG	94	114,625	*Dra* I
SNP30	GGGCTCTCTTGCGCCTATATT	CAGCTTATATAAATAAAAATTAGC	95	123,140	*Hin* fI
RAPD marker					
A07	GATGGATTTGGG		800		
A20	TTGCCGGGACCA		1100, 800		
A22	TCCAAGCTACCA		1520		
A23	AAGTGGTGGTAT		1200		
A26	GGTGAGGATTCA		1400		
A39	CCTGAGGTAACT		2027		
A41	TGGTAGGTAACT		930		
A57	ATCATTGGCGAA		800		
B15	CCTTGGCATCGG		600		
B68	CACACTCGTCAT		1078		
B71	GGACCTCCATCG		1220		
B84	CTTATGGATCCG		700, 600, 550		
B86	ATCGAGCGAACG		1350		
B96	CTGAAGACTATG		850, 750		
B99	TTCTGCTCGAAA		1400		
C00	GAGTTGTATGCG		1350		
SSR marker					
CMBR 12	ACAAACATGGAAATAGCTTTCA	GCCTTTTGTGATGCTCCAAT	134		
CMBR 22	TCCAAAACGACCAAATGTTCC	ATACAGACACGCCTTCCACC	177		
CMBR 53	GCCTTTTGTGATGCTCCAAT	AAACAAACATGGAAATAGCTTTCA	134		
CMBR 83	CGGACAAATCCCTCTCTGAA	GAACAAGCAGCCAAAGACG	142		
CMBR 120	CTGGCCCCCTCCTAAACTAA	CAAAAAGCATCAAAATGGTTG	167		
CMN 04-07	GAAAGCATTAAATATGGCATTGG	AAGCTTAACAGCTTCCAGGG	286		
CMN 04-40	CACCTGACGATAGGGGTGTT	AGTATTCGGGTTGGCAAAAA	212		
CMN 08-22	CATCCTCCTCATCCTCCTCA	ACGGATGAATCGGAACTTCA	223		
CMN 08-90	CCACGCCCTCTATACCCATA	GGGACTGTTGGGTTTTCTGA	210		
CMN 21-41	GAGGAAATTTTGGAGTTTTTCAA	TTCCAGACATCTAAAGGCATTG	281		
CMN 22-16	CAGAGGAGGTGGAACTAACCA	CCATTTTCAACCTCCCAAGA	233		

[a] SNP names refer to Tanaka et al. (2013).

[b] R = A or G, K = T or G. Lowercase alphabetic characters in primer sequences indicate mismatch sites for both wild and mutant sequences.

[c] For the chloroplast genotyping markers, PCR product size and position refer to the melon chloroplast genome sequence (accession No. JF412791.1).

Supplemental Table 4 Cytoplasm type in Kazakh melon accessions inferred by PCR amplification of five chloroplast genome markers and their corresponding sequences. genome markers their corresponding sequences

Cytoplasm[a] type	No. of Kazakh melon accessions	InDel1		SNP2		SNP18		SNP19		SNP30	
		Fragment type	Sequence type[a]	Fragment type	Sequence[a]	Fragment type	Sequence[a]	Fragment type	Sequence[a]	Fragment type	Sequence[a]
Ia	2	339 bp	Non-deletion	Non-digested	C	Non-digested	A	Digested	A	Non-digested	T
Ib-1/-2	52	339 bp	Non-deletion	Digested	A	Digested	T	Non-digested	G	Non-digested	T
Ib-3	33	334 bp	Deletion	Digested	A	Digested	T	Non-digested	G	Non-digested	T
Ic	0	339 bp	Non-deletion	Non-digested	C	Non-amplified[2]	T	Non-digested	G	Digested	C

[a] Cytoplasm names and InDel and SNP sequences are based on the study by Tanaka et al. (2013).

[b] PCR amplification was not successful in Ic-type melons because of the high number of mismatched bases in the forward primer binding site.
